# Parameter optimisation for mitigating somatosensory confounds during transcranial ultrasonic stimulation

**DOI:** 10.1016/j.brs.2025.06.009

**Published:** 2025-06-11

**Authors:** Benjamin R. Kop, Linda de Jong, Butts Pauly Kim, Hanneke E.M. den Ouden, Lennart Verhagen

**Affiliations:** a Donders Institute for Brain, Cognition, and Behaviour, Radboud University, Thomas van Aquinostraat 4, 6525 GD, Nijmegen, the Netherlands; b Department of Radiology, Stanford University, 300 Pasteur Drive, Stanford, CA, USA

**Keywords:** Transcranial ultrasonic stimulation (TUS), Neuromodulation, Peripheral confounds, Somatosensory confounds, Experimental design & control, Peripheral nervous system

## Abstract

**Background:**

Transcranial ultrasonic stimulation (TUS) redefines what is possible with non-invasive neuromodulation by offering unparalleled spatial precision and flexible targeting capabilities. However, peripheral confounds pose a significant challenge to reliably implementing this technology. While auditory confounds during TUS have been studied extensively, the somatosensory confound has been overlooked thus far. It will become increasingly vital to quantify and manage this confound as the field shifts towards higher doses, more compact stimulation devices, and more frequent stimulation through the temples where co-stimulation is more pronounced.

**Methods:**

Here, we provide a systematic characterisation of somatosensory co-stimulation during TUS. We also identify the conditions under which this confound can be mitigated most effectively by mapping the confound-parameter space. Specifically, we investigate dose-response effects, pulse shaping characteristics, and transducer-specific parameters.

**Results:**

We demonstrate that somatosensory confounds can be mitigated by avoiding near-field intensity peaks in the scalp, spreading energy across a greater area of the scalp, ramping the pulse envelope, and delivering equivalent doses via longer, lower-intensity pulses rather than shorter, higher-intensity pulses. Additionally, higher pulse repetition frequencies and fundamental frequencies reduce somatosensory effects. Through our systematic mapping of the parameter space, we also find preliminary evidence that particle displacement (strain) may be a primary biophysical driving force behind peripheral somatosensory co-stimulation.

**Conclusion:**

This study provides actionable strategies to minimise somatosensory confounds, which will support the thorough experimental control required to unlock the full potential of TUS for scientific research and clinical interventions.

## 1. Introduction

Transcranial ultrasonic stimulation (TUS) redefines the limits of non-invasive neuromodulation with its unprecedented spatial resolution and targeting capabilities [1–11]. However, peripheral co-stimulation poses a significant challenge to the reliable application of this technology. Peripheral effects such as somatosensation increase participant burden, can result in false inferences [12–16], and contribute to the substantial placebo effects observed during brain stimulation [14,17–19]. Stringent experimental control is therefore required to infer direct neuromodulatory contributions to observed effects. While auditory confounds during TUS have been studied extensively [14,20–27], possible somatosensory confounds remain unexplored. As TUS is increasingly applied at higher doses, with compact stimulation devices, and over the temples [28–33], it will be critical to effectively manage somatosensory co-stimulation to ensure validity and specificity in this rapidly advancing field.

When an ultrasound beam is focused directly on the peripheral nervous system (PNS), such as the fingertip, tactile sensations can be felt. Here, ultrasound stimulates mechanoreceptors in the skin, including Merkel cells, Ruffini endings, Meissner corpuscles, and Pacinian corpuscles, which predominately innervate A-β fibres [34–38]. At higher doses, thermal and nociceptive sensations emerge, likely driven by the recruitment of higher threshold mechanoreceptors that innervate Type 1 A-δ and C fibres [34,37,39–42]. Peripheral somatosensation of ultrasound relies on mechanosensitive ion channels, including TRPV1, TRPA1, TREK-1, TRAAK, and Piezo channels. These channels not only play a critical role in the biological mechanism underlying TUS in the PNS, but are similarly implicated in TUS neuromodulation in the central nervous system (CNS) [39,43–50].

The parameters of the ultrasound stimulation, such as the fundamental frequency and pulse repetition frequency, influence somatosensation. For instance, lower fundamental frequencies elicit stronger sensations [34–37,41,42,51–53]. Notably, certain parameters such as fundamental frequency differ in their relative strength of key biophysical effects like particle displacement and acoustic radiation force (ARF). Therefore, parameter mapping studies provide a valuable opportunity to elucidate the primary biophysical mechanisms underlying ultrasonic neuromodulation [34]. However, studies using peripherally focused ultrasound typically employ stimulation parameters distinct from those used during TUS, and do not fully explore the conditions relevant in the context of somatosensory co-stimulation during transcranial neuromodulation. Therefore, in the present study we investigate peripheral somatosensory effects across parameters relevant specifically to transcranial ultrasound for neuromodulation of the human brain.

In this pre-registered [54] study, we bring TUS somatosensory confounds into focus by qualitatively characterising their nature and systematically mapping the confound-parameter space to identify avenues to minimise their impact. To ensure sufficient sensitivity to detect the effects of manipulating stimulation parameters, we intentionally operate under conditions that we expect will amplify somatosensory confounds. We further leverage this systematic investigation to explore the primary biophysical mechanisms of TUS that drive neuromodulation and provide preliminary evidence of particle displacement as a central biophysical mechanism. By putting forward actionable strategies to mitigate somatosensory confounds, we equip researchers with tools to optimise TUS studies for minimal burden and high inferential power, thus advancing TUS towards reliable and impactful applications across scientific, commercial, and clinical settings.

## 2. Methods

### 2.1. Participants

Twenty-nine participants were recruited, and twenty-five participants completed the study (14 female, 11 male, aged 25 ± 4.3). Three participants were excluded after MRI intake, because the target sample size was achieved. One participant was excluded for psychological distress unrelated to TUS. All participants were free of psychiatric and neurological disorders, had no contraindications to brain stimulation, and provided informed consent. The study was approved by the Radboud University faculty of Social Sciences ethics committee (ECSW-2024-085) and conducted in accordance with the Declaration of Helsinki.

### 2.2. MRI

Both T1w and ultra-short echo time (UTE) MRI scans were acquired for each participant to support TUS neuronavigation and acoustic simulations [55,56]. See Supplementary Table 2 for sequence specifications.

### 2.3. Transcranial ultrasonic stimulation (TUS)

**Fig. 1.**
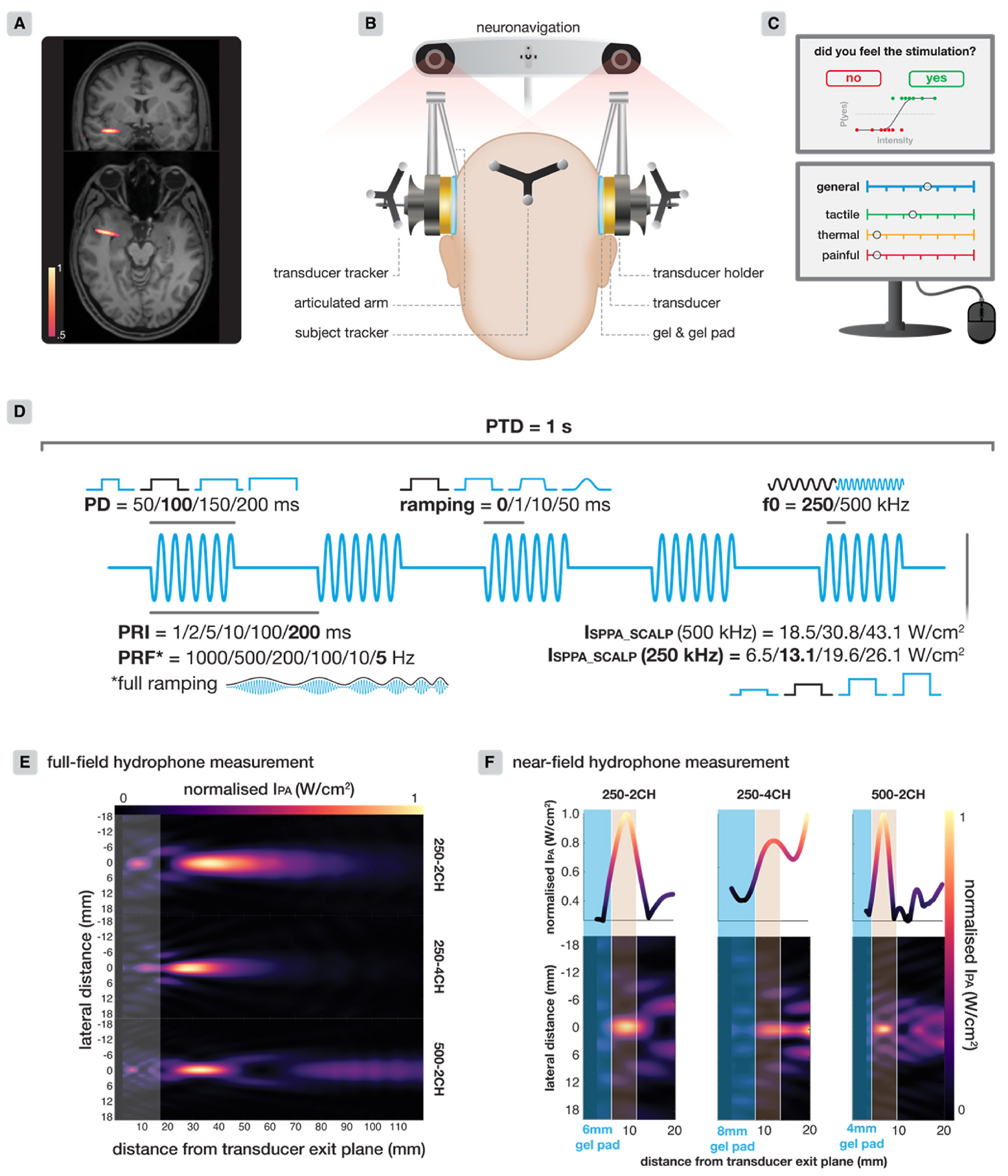
TUS experimental setup and methodology. **(A)** Representative acoustic simulation of temporal lobe white matter targeting, depicting the min-max normalised −3dB (full-width half-maximum) intensity profile for 250 kHz stimulation. **(B)** Experimental setup. **(C)** Experimental task. Top: yes/no questions used to estimate sensory thresholds (psychometric curve not visible to participant). Bottom: visual analogue scales. First, the overall holistic experience of somatosensory co-stimulation is captured with the ‘general’ VAS. Here, we determine whether TUS was felt only slightly, or very strongly. Next, subscales for tactile, thermal, and painful sensations capture the constituent sensory components specifically. **(D)** TUS protocol. Manipulated parameters are noted, with the standard protocol depicted in bold/black. Each parameter is manipulated separately while the other parameters remained standard with one exception: when investigating different PRFs, full ramping was applied at each level. **(E)** Full-field hydrophone measurements for each transducer to quantify the intended transcranial acoustic field. The highlighted band reflects the transducer near-fields. **(F)** Near-field higher-resolution hydrophone measurements for each transducer, used to equalise exposure in the scalp. Gel-pad thickness (blue) for each transducer and the scalp (beige) are depicted. The gel-pad thicknesses, focal depths, and absolute stimulation intensities were adjusted such that the integrated maximum/total intensity in the scalp was equalised between transducers (see Supplementary Figs. 1–2 for details).

TUS was delivered using two NeuroFUS systems (supplier/support: BrainBox Ltd., Cardiff, UK; manufacturer: Sonic Concepts Inc., Bothell, WA, USA). Each of the four-channel radiofrequency amplifiers powered one piezoelectric transducer via an electrical impedance matching network. The experiment involved three transducers: a two-element 250 kHz transducer (250-2CH; serial number: CTX250-014, aperture diameter *d* = 45 mm, area = 15.90 cm^2^), a two-element 500 kHz transducer (500-2CH; serial number: CTX500-006, *d* = 45 mm, area = 15.90 cm^2^), and a four-element 250 kHz transducer (250-4 CH; serial number: CTX250-026, *d* = 64 mm, area = 33.18 cm^2^; Fig. 1E). Detailed specifications for each transducer along with hydrophone measurements are reported in Supplementary Figs. 1–2 and Supplementary Table 1, in line with ITRUSST Standardised Reporting Guidelines [57]. Transducer performance was monitored across sessions [58,59].

Transducers were coupled to the scalp using ultrasound gel [59] and a gel-pad (Aquasonic 100 & Aquaflex, Parker Laboratories, NJ, USA). Prior to coupling, the participant’s hair around the stimulation site was prepared with ultrasound gel. Gel-pad thicknesses were 6, 8, and 4 mm for 250-2 C H, 250-4 C H, and 500-2 C H transducers respectively, to optimise coherence of intensities in the scalp between transducers (Fig. 1F; see Supplementary Figs. 1–2 for details).

Transducer position was determined and maintained during the experiment by means of individualised neuronavigation based on participants’ T1w MRI scans (Localite GmbH, Sankt Augustin, Germany). TUS was targeted at the white matter of the temporal lobe, which is an inactive site within the context of the present study that is not expected to either produce or interact with sensory perception. A representative post-hoc acoustic simulation shows that temporal white matter targeting was successful (Fig. 1A; see Supplementary Fig. 3 for simulation methodology).

During the TUS experiment, two transducers were positioned bilaterally over the temporal window and held in place mechanically by articulated arms fastened to scaffolding built around the participant chair (Fig. 1B). A chin rest ensured that the participant was held firmly in place. Only one transducer administered stimulation per trial. To control for any putative difference in sensitivity to peripheral co-stimulation between the two sides of the head confounding observed differences between conditions, the transducer sides were switched halfway through the experiment, with the initial side counterbalanced between participants.

### 2.4. Somatosensory outcome measures

We quantified participants’ experience of TUS peripheral somatosensory co-stimulation through continuous visual analogue scales (VAS) and sensory thresholds. VAS ratings ranged from 0 (no sensation) to 10 (very intense sensation). First, participants provided an overall rating of their somatosensory experience of TUS (i.e., the ‘general’ rating scale). This initial rating captures the holistic perception of somatosensory co-stimulation intensity. Next, to gain more insight into the nature of the somatosensory effects, participants separately rated three subscales for tactile, thermal, and painful sensations specifically. This approach allowed us to capture both the overall experience of somatosensation, as well as to independently evaluate the nature of the sensations (Fig. 1C).

Sensory thresholds were measured by asking participants whether they could perceive a given protocol (yes/no) administered at various intensities. A custom thresholding procedure was used building on the parameter estimation by sequential testing method [60,61] (see Supplementary Fig. 4 for details). The threshold was defined as the TUS intensity at which a fitted psychometric curve predicted a 50 % likelihood of perception.

**Fig. 2.**
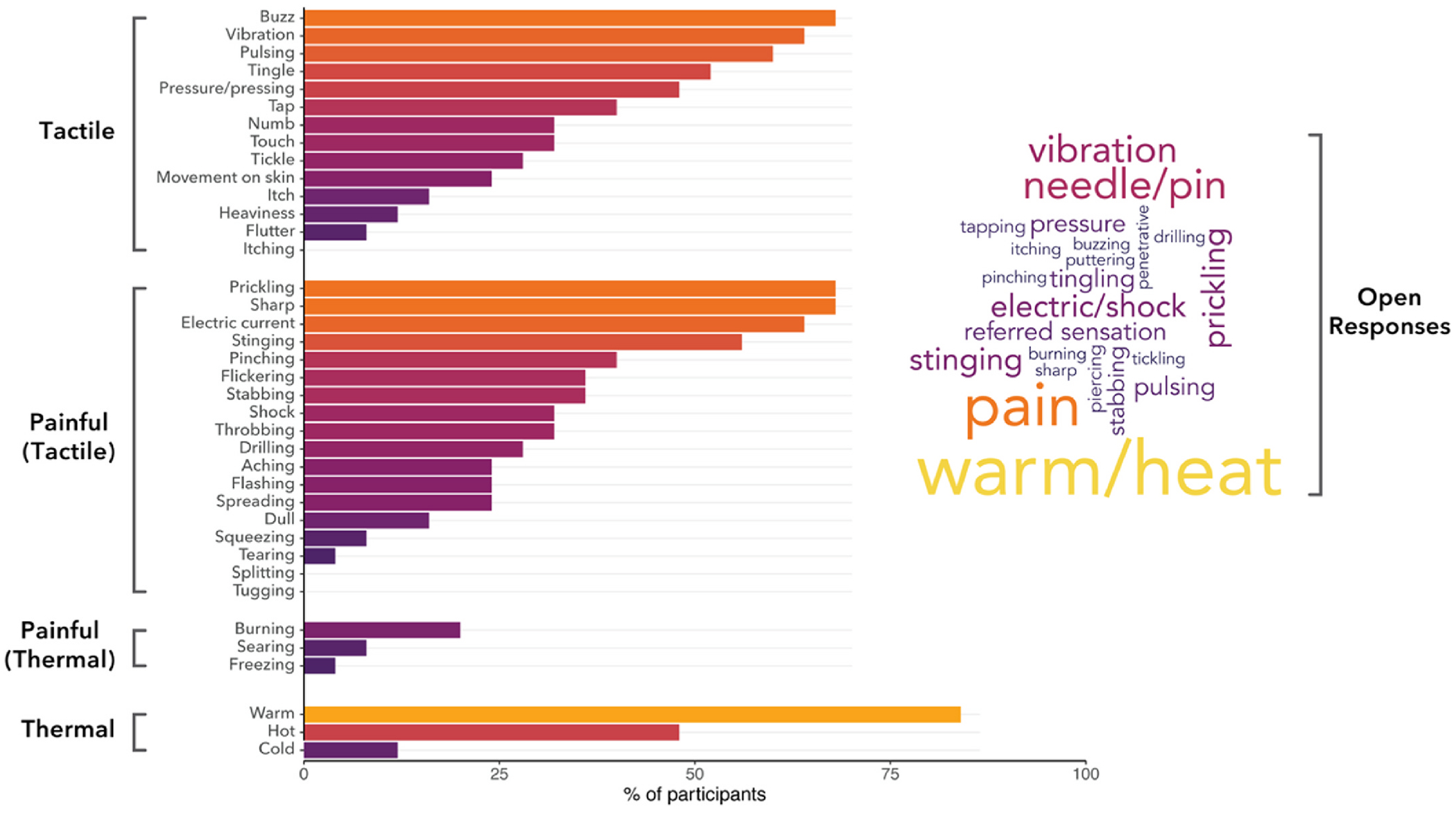
Characterisation of peripheral somatosensation during TUS. Descriptors were acquired via questionnaires completed after the main experiment. Here, participants retrospectively reported on all sensations they experienced across the entire session, encompassing all administered protocols collectively. Bars depict the percentage of participants who reported a given sensation on a closed psychometric questionnaire. The word cloud depicts descriptors mentioned in response to an open question, with size reflecting the frequency of a given descriptor.

At the end of the experiment, we qualitatively characterised the somatosensory confound. Participants first responded to an open question prompting them to describe the sensations they experienced throughout the study. They then completed an adapted closed-format psychometric questionnaire for reporting somatosensory percepts [62] (Fig. 2).

### 2.5. Study design

A sham-controlled, double-blind online TUS design was implemented, incorporating inter-subject trial-level counterbalancing. Full details on counterbalancing and task structure are provided in Supplementary Fig. 5.

The sham condition involved an auditory stimulus played over speakers, also present during TUS trials as an auditory mask. The volume was set uniformly across participants and was experienced as quite loud but not intolerable. This sound was designed to replicate the experiential qualities of our TUS protocols as closely as possible. Both the auditory stimulus and the code to generate it are publicly available here: https://doi.org/10.5281/zenodo.14052159. The PsychoPy [63] IDE for Python was used to administer sham/auditory masking and TUS, set TUS parameters, and to record participants’ responses.

The standard TUS protocol (Fig. 1D, **bold**) was applied using transducer 250-2 C H at a 250 kHz fundamental frequency (*f_0_*) with a square wave pulse repetition frequency (PRF) of 5 Hz, pulse repetition interval (PRI) of 200 ms, a pulse duration (PD) of 100 ms, a duty cycle (DC) of 50%, and a pulse train duration (PTD) of 1 s. The spatial-peak pulse-average intensity (I_SPPA_) was 19.72 W/cm^2^, and the corresponding near-field intensity in the scalp (I_SPPA.SCALP_) was 13.06 W/cm^2^. An inter-trial interval of approximately 10 s was used. All conditions adhered to ITRUSST recommendations for biophysical safety [64] (see Supplementary Table 1 for safety metrics). To identify strategies to mitigate the somatosensory confound, we investigated multiple facets of this standard protocol, as well as transducer characteristics. Each section below describes a different investigation.

#### 2.5.1. Dose & dose modality (intensity/pulse duration)

We heuristically defined dose as exposure, that is, the integrated spatial-peak pulse-average intensity in the scalp (^∫^ *I_SPPA.SCALP_*) over the PTD. While recent discussions in the field distinguish between exposure and absorbed, equivalent, and effective dose [65] – each accounting for interactions with biological tissue – we use the broader term ‘dose’ here for simplicity. Dose is given by the formula:

$$\mathrm{Dose}=\mathrm{PD}\cdot \mathrm{PRF}\cdot \mathrm{PTD}\cdot I_{\mathrm{SPPA.SCALP}}$$

We applied stimulation at four doses: 3.3/6.5/9.8/13.1 J/cm^2^. The same dose was achieved via manipulation of two ‘dose modalities’: I_SPPA.__SCALP_ and pulse duration (PD). We included dose modality to determine whether somatosensory co-stimulation was influenced by ‘dose sharpness’, i.e., the speed of equal dose delivery through shorter and higher intensity pulses versus longer and lower intensity pulses. Here, the interaction between ‘Dose’ and ‘Dose Modality’ (I_SPPA.SCALP_/PD) can yield insight into whether increasing intensity versus pulse duration has a different effect on the magnitude of somatosensory co-stimulation. Levels for I_SPPA.SCALP_ were 6.5/13.1/19.6/26.1 W/cm^2^, with PD = 100 ms held constant (‘PD _100 ms_’). Levels for pulse duration were 50/100/150/200 ms, with I_SPPA.SCALP_ = 13.1 W/cm^2^ held constant (see Fig. 1D).

#### 2.5.2. Amplitude modulation (ramping)

We determined the effect of ramping on sensory thresholds by comparing square-wave modulated TUS with tapered cosine ramped amplitude modulation durations of 1 ms (0.01 × PD_100 ms_), 10 ms (0.1 × PD_100 ms_), and 50 ms (0.5 × PD_100 ms_; maximum ramping).

#### 2.5.3. PRF

PRFs were administered at 5/10/50/100/200/500/1000 Hz, covering the range of typically applied PRFs in the human literature to date [3,11,14,66]. Here, amplitude modulation consisted of a fully smoothed Tukey ramp (PD = PRI; tapered cosine ramp duration = 0.5·PRI), creating a more narrowband frequency distribution for the administered PRF, in contrast to the wider frequency distribution of square-wave pulse envelopes.

#### 2.5.4. Fundamental frequency (f_0_)

Stimulation was applied at 250 and 500 kHz using two transducers (250-2 C H & 500-2 C H). Focal depths, intensities, and gel-pad thicknesses were adjusted to optimise comparability and account for varying intensity (distribution) in the scalp between the two transducers (see Figs. 1F and 5). The dose-response relationship was mapped for 500 kHz, similar to 250 kHz, by manipulating the I_SPPA.SCALP_ (18.5/30.8/43.1 W/cm^2^; Supplementary Fig. 8).

#### 2.5.5. Transducer aperture area

The impact of aperture area on somatosensory co-stimulation was investigated by comparing two transducers with aperture areas of 15.90 and 33.18 cm^2^ (250-2 C H & 250-4 C H), each applying the same integrated total intensity to the scalp. The larger aperture transducer spread this energy over a wider area, thus reducing the intensity per unit area.

#### 2.5.6. Near-field peak amplitude

The annular arrays commonly used in human TUS research can produce near-field intensity peaks when the focus is steered axially. We quantified the impact of these near-field peaks on peripheral somatosensory co-stimulation by applying our standard TUS protocol at focal depth settings of 35.7, 38.3, 40.3 (standard), 42.1, and 44.1 mm. These focal depths were applied uniformly across participants and refer to the distance between the exit plane of the transducer and the centre of the full-width half-maximum intensity profile. These depths corresponded to manufacturer-reported near-field peak intensities in the scalp of 5.3, 9.4, 13.8, 17.9, and 22.3 W/cm^2^, respectively.

#### 2.5.7. Temporal summation

We additionally examined whether somatosensory co-stimulation changes progressively throughout an online experiment by assessing inter-trial changes in VAS ratings. Each trial consisted of TUS at a 1-s PTD, after which participants’ provided VAS ratings. Trials repeated approximately every 10 s. To assess temporal changes in VAS ratings, we tested three scenarios. First, in the ‘interspersed’ approach, we applied the standard protocol once every 6 trials. Second, in the ‘sequential’ approach, we applied this standard protocol for 6 consecutive trials. Finally, we assessed temporal changes in VAS ratings across four ‘blocks’. These blocks were composed of multiple protocols that were counterbalanced between participants but repeated within participants. This design allowed us to assess potential temporal summation of somatosensory co-stimulation across multiple TUS protocols simultaneously (see Supplementary Fig. 5 for a more detailed overview of trial structure).

To investigate possible sustained effects outlasting the stimulation period, as relevant for offline protocols with their longer pulse train durations (PTDs), we extended the PTD to 10 s at an I_SPPA.SCALP_ of 5.23 W/cm^2^. Participants continuously reported their sensations on a VAS throughout this extended PTD, capturing the onset, development, and persistence of somatosensory co-stimulation in response to sustained stimulation.

### 2.6. Analysis

Data were processed, visualised, and analysed with R (v4.4.0). Data and code to reproduce the results have been open-sourced here: https://doi.org/10.34973/z3rz-jt33. Sham-correction was performed by subtracting the average VAS rating for sham trials from each trial-level VAS rating per participant (see Supplementary Fig. 6 for sham). Linear mixed models (LMMs) were fitted to assess main effects and interactions for manipulated parameters on absolute VAS ratings and sensory thresholds, typically with a maximal random effects structure [67]. These models were implemented through the lme4 [68] package in R. Statistical significance was set at a two-tailed α = 0.05 and computed with t-tests using the Satterthwaite approximation of degrees of freedom.

For visualisation, VAS ratings were z-score normalised per participant to remove between-subject individual differences in intercept and range. These normalised data were used exclusively for visualisation to match the analyses they represent, as the linear mixed models we employed also account for this inter-individual variability.

## 3. Results

All participants reported feeling tactile, thermal, and painful sensations during the experiment. One participant experienced psychological distress unrelated to TUS and discontinued participation; their data was not analysed. Another participant displayed skin irritation at the stimulation site after participation, which resolved within a few hours. There were no further adverse events.

### 3.1. Qualitative characterisation of the somatosensory confound

On a closed psychometric questionnaire, more than half of participants reported warmth, buzzing, prickling, sharpness, electric current, vibration, pulsing, stinging, and tingling (Fig. 2). In response to an open question, the most prevalent sensations were warmth/heat, pain, a needle/pinprick, prickling, vibration, and electric current/shocks (Fig. 2). These sensations were measured after completion of the main experiment and therefore pertain to sensory experiences across the entire experiment. The co-occurrence of these sensations is depicted in Supplementary Fig. 7.

This somatosensory co-stimulation likely arises from direct stimulation of mechanoreceptors and sensory fibres, which are present in greater density at the temples as compared to the top of the scalp. TUS applied over the temples may additionally engage trigeminal ganglion cell bodies. Indeed, two participants reported referred sensations to their teeth and nose, respectively. All subsequent results we discuss pertain to the sensory thresholds and trial-by-trial VAS ratings (general/tactile/thermal/painful) during the main experiment.

**Fig. 3.**
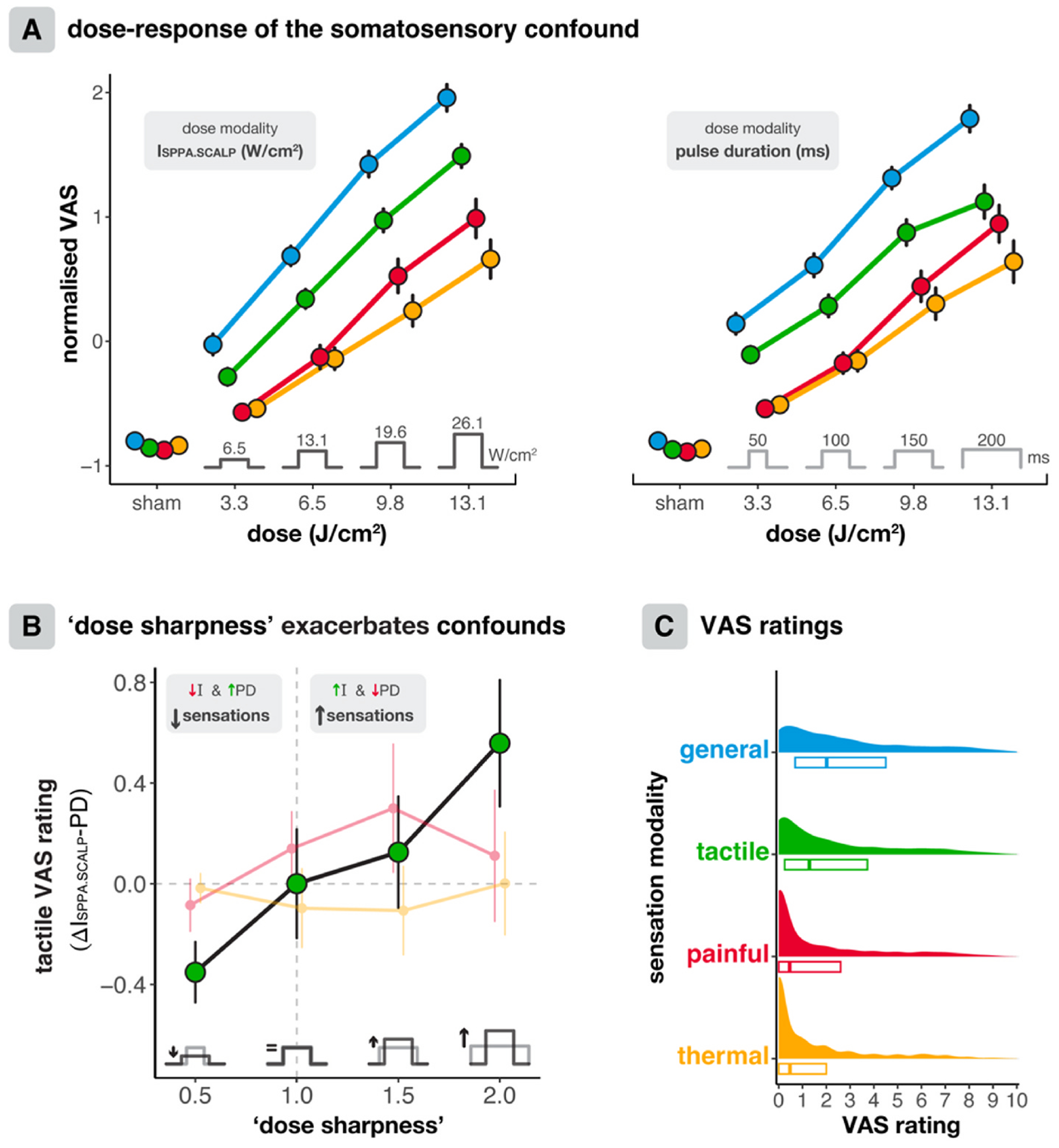
Dose-response of somatosensory confounds. **(A)** Dose-response of the somatosensory confound (250 kHz). Peripheral sensations become stronger as dose increases, both when increasing dose via intensity (left) and pulse duration (right) modalities. Tactile sensations are felt the earliest and the strongest. At higher doses, painful sensations become significantly stronger than thermal sensations. Points represent mean z-scored VAS ratings, error bars depict the standard error. Absolute VAS ratings are provided in Supplementary Fig. 10. **(B)** For tactile sensations specifically, higher ‘dose sharpness’ elicits stronger sensations. That is, shorter, higher intensity pulses cause more tactile sensations than longer, lower intensity pulses. Data reflect the absolute difference in VAS rating for the darker pulse waveform compared to the lighter pulse waveform. **(C)** Distribution of absolute VAS ratings across all conditions of the experiment, including participant ratings for the magnitude of co-stimulation they felt overall (i.e., general), as well as subscales for tactile, painful, and thermal sensations.

Tactile sensations were rated significantly higher on the VAS than thermal and painful sensations for each applied intensity (Fig. 3; all *p* < 0.001). Thermal and painful sensations did not differ significantly at lower doses (i.e., 3.3 and 6.5 J/cm^2^), but painful sensations became significantly more salient than thermal sensations at higher doses (i.e., 9.8 and 13.1 J/cm^2^, all *p* < 0.001, Fig. 3A), potentially resulting from hyperactivation of receptor structures including those innervating A-β fibres [51,69], or from central prioritisation of pain processing.

### 3.2. Dose-response relationship of somatosensory confounds

For both 250 and 500 kHz TUS, linear mixed models revealed that higher doses resulted in more peripheral somatosensation, as quantified by VAS ratings. At 250 kHz *f_0_*, four dose levels were tested (Fig. 3B; 3/6/9/12 J/cm^2^). Dose was manipulated along two modalities, by increasing either intensity or pulse duration. A three-way LMM with a random intercept for Dose, Dose Modality, and Sensation Modality revealed a significant three-way interaction (*F*(2,4662) = 5.91, *p* = 0.003, ${\text{η}}_{\text{p}}^{2}=0.003$). Follow-up two-way LMMs with Dose and Dose Modality as predictors all revealed a significant main effect of Dose (all *p* < 0.0001). At a 500 kHz fundamental frequency (*f_0_*), there was a significant effect of Dose, manipulated solely though I_SPPA. SCALP_, for each Sensation Modality (Supplementary Fig. 8; all *p* < 0.001).

While these findings show that reducing dose can ameliorate the somatosensory confound across fundamental frequencies, it also poses a risk of diminishing the intended central nervous system neuromodulation. Importantly, our experiments also revealed opportunities to minimise the somatosensory confound while maintaining dose. For example, we found that ‘dose sharpness’, quantified as the ratio of peak intensity to duration at equivalent dose, predicts tactile somatosensory co-stimulation, which is the most prominent (Fig. 3B). Here, at equivalent doses, interactions reveal that shorter and higher intensity pulses cause more tactile somatosensation than longer and lower intensity pulses (Dose x Dose Modality: *F* (1,1522.2) = 15.5, *p* < 0.0001, ${\text{η}}_{\text{p}}^{2}=0.01$), with follow-up LMMs showing significant differences between Dose Modality for the lowest and highest conditions (Dose = 3.3: *F* (1,374) = 7.03, *p* = 0.008, ${\text{η}}_{\text{p}}^{2}=0.018$; Dose = 13.1: *F* (1,373) = 10.3, *p* = 0.001, ${\text{η}}_{\text{p}}^{2}=0.027$). We note that this relationship was significant for tactile sensations but was absent for painful and thermal sensations. Here, dose sharpness is experienced as ‘tapping’ rather than pain or heat. Thus, tactile sensations can be minimised while maintaining dose by favouring longer pulses with lower intensities over short pulses with higher intensities.

### 3.3. Pulse shaping & temporal characteristics

#### 3.3.1. Amplitude modulation (ramping)

**Fig. 4.**
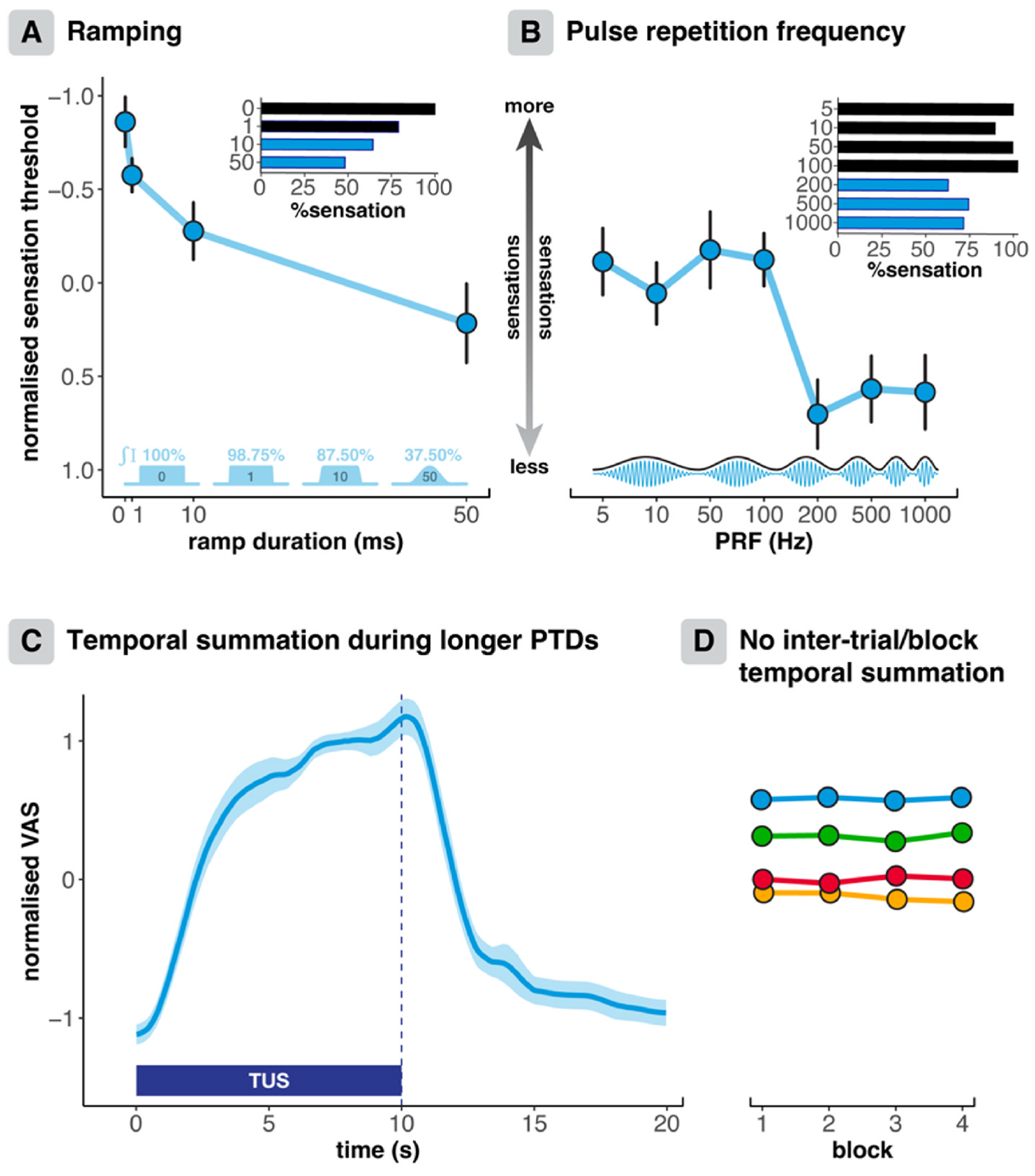
Pulse shaping & temporal characteristics. **(A)** Ramping for 10 and 50 ms significantly reduced the somatosensory confound. Normalised thresholds are depicted on a flipped y-axis, where visually higher points reflect stronger sensations (i.e., lower thresholds). The histogram depicts the average threshold as a percentage, and the pulse envelopes illustrate the integrated intensity as a percentage, both as compared to a square-wave pulse. **(B)** Higher pulse repetition frequencies (PRFs) elicited significantly less somatosensory co-stimulation than lower PRFs. **(C)** After a sharp initial incline, somatosensory co-stimulation increases steadily during a 10 s PTD, indicating that somatosensory confounds may develop over the longer PTDs typically used in offline protocols. **(D)** Somatosensory co-stimulation remains constant across repeated sets (‘blocks’) of protocols, demonstrating that there is no inter-trial temporal summation of somatosensory co-stimulation in this online protocol. Additionally, there was no inter-trial temporal summation for identical protocols applied consecutively, nor when interspersed throughout the experiment (see Supplementary Fig. 9). Points represent condition means and error bars depict the standard error.

Ramping significantly decreased the somatosensory confound (Fig. 4A; *F* (3,72) = 8.46, *p* < 0.0001, ${\text{η}}_{\text{p}}^{2}=0.261$), resulting in less sensation in response to 10 and 50 ms of tapered cosine amplitude modulation compared to square wave modulation (10 ms: *p* = 0.023; 50 ms: *p* < 0.0001; FDR corrected for multiple comparisons). In line with our findings for ‘dose sharpness’, this result suggests that the gradient of change in TUS amplitude may contribute to tactile peripheral co-stimulation.

#### 3.3.2. Pulse repetition frequency (PRF)

Pulse repetition frequencies of 100 Hz and lower were associated with more peripheral somatosensation (i.e., lower thresholds) than higher pulse repetition frequencies (Fig. 4B). There was a significant main effect of PRF on thresholds (*F* (6,144) = 4.10, *p* = 0.001, ${\text{η}}_{\text{p}}^{2}=0.146$; see Supplementary Table 3 for post-hoc paired comparisons). Sensations for grouped PRFs of 5, 10, 50, and 100 Hz were significantly lower than for PRFs of 200, 500, and 1000 Hz (*F* (1,24) = 13.6, *p* = 0.001, ${\text{η}}_{\text{p}}^{2}=0.361$). These results suggest that peripheral sensory nerves are preferentially activated by neurophysiologically relevant PRFs within their endogenous firing rates.

#### 3.3.3. Temporally summative somatosensory co-stimulation

##### 3.3.3.1. No inter-trial cumulation of somatosensory confounds in an online paradigm.

VAS ratings remained stable throughout this online experiment for a single stimulation protocol interspersed throughout a 10-min stimulation period (Trial: *F* (1,24) = 0.061, *p* = 0.808, ${\text{η}}_{\text{p}}^{2}=0.003$, BF01 = 30.8), and for six successive trials of the same protocol (*F* (1,24) = 0.427, *p* = 0.52, ${\text{η}}_{\text{p}}^{2}=0.017$, BF01 = 28.1; see Supplementary Fig. 9). These results also demonstrate the consistency of the VAS ratings as an outcome measure. When assessing blocks of trials in which the same set of protocols were administered, VAS ratings also remained consistent over time (*F* (1,24) = 1.63, *p* = 0.214, ${\text{η}}_{\text{p}}^{2}=0.064$, BF01 = 283.6). In this specific but representative online experimental paradigm (ITI = 10 s, PTD = 1 s), there was no inter-trial cumulation of the somatosensory confound.

##### 3.3.3.2. Cumulation of somatosensory co-stimulation for offline paradigms.

Offline TUS protocols are typically characterised by longer pulse train durations where somatosensory co-stimulation may develop as stimulation progresses. Here, we show that following participants’ initial response to peripheral co-stimulation, sensations continue to build steadily until the stimulation ends, whereafter sensations subside almost immediately. Some minor sensory effects persist for a few seconds before returning fully to baseline.

### 3.4. Transducer-specific characteristics

#### 3.4.1. Fundamental frequency & biophysical mechanisms

**Fig. 5.**
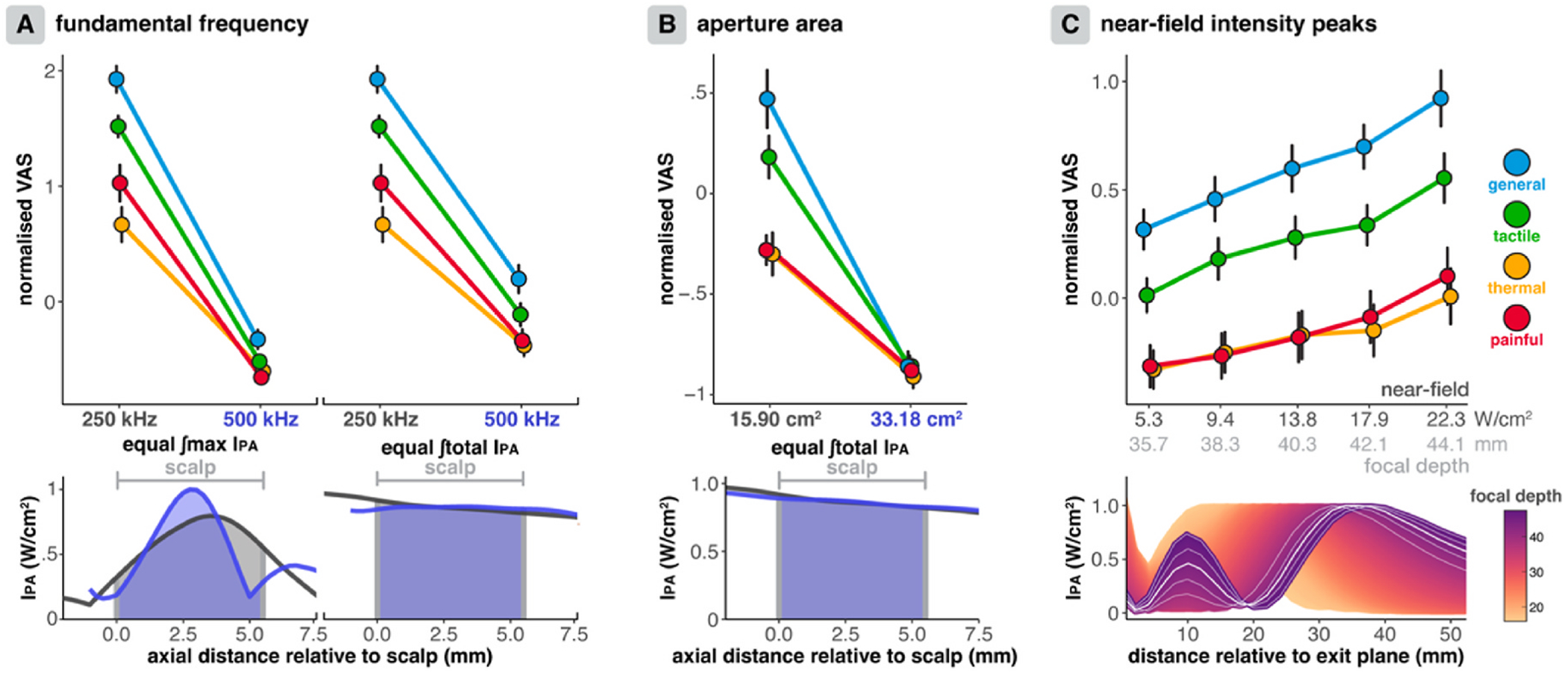
Transducer-specific parameters. **(A)** Higher fundamental frequencies elicit significantly less somatosensory co-stimulation, both when the integrated maximum intensity (bottom left) and integrated total intensity (bottom right) in the scalp are equalised. **(B)** A larger aperture area transducer delivering an equal integrated total intensity (bottom) also elicited less co-stimulation. **(C)** Higher magnitude near-field intensity peaks caused by axial steering to larger focal depths for this transducer elicited more somatosensory co-stimulation. On the lower panel, white lines indicate the manufacturer axial profile measurements for the applied focal depths. Points indicate conditions means and error bars depict standard error.

TUS at a 500 kHz *f_0_* elicited significantly less somatosensory co-stimulation than a 250 kHz *f_0_*, regardless whether the maximum or total integrated intensity in the scalp was equalised (Fig. 5A; ∫max.: *F* (1,24) = 97.6, *p* < 0.0001, ${\text{η}}_{\text{p}}^{2}=0.803$; ∫total: *F* (1,24) = 58.7, *p* < 0.0001, ${\text{η}}_{\text{p}}^{2}=0.71$). Therefore, increasing *f_0_* can decrease somatosensory confounds.

We note that the direction of this relationship generates the hypothesis that particle displacement may be a primary biophysical mechanism driving peripheral neuromodulation, as this biophysical effect is stronger at lower fundamental frequencies [70]. To further explore this possibility, we investigated whether somatosensory co-stimulation scaled with pressure (~particle displacement) or intensity (~ARF). Specifically, we compared non-nested LMMs using intensity (*I*) or pressure ($\sqrt{I}$) as a predictor. Across all Sensory Modalities, we found that pressure was *>*90 % likely to better explain the variance in our data than intensity (general: ΔAIC = 10.8, *w* = 0.995; tactile: ΔAIC = 9.7, *w* = 0.992; thermal: ΔAIC = 5.9, *w* = 0.949; painful: ΔAIC = 4.9, *w* = 0.921). Taken together, these results provide preliminary, hypothesis-generating evidence that particle displacement may be a more relevant biophysical force than ARF in eliciting peripheral somatosensory effects. This hypothesis requires confirmation through more direct biophysical measurements.

#### 3.4.2. Transducer aperture area

A larger aperture area four-element annular array transducer (33.18 cm^2^) elicited significantly less somatosensory co-stimulation than a smaller two-element transducer (15.90 cm^2^) when equalising the integrated total intensity in the scalp (Fig. 5B; *F* (1,24) = 40.5, *p <* 0.0001, ${\text{η}}_{\text{p}}^{2}=0.628$). Decreasing the intensity per unit area in the near-field using larger aperture transducers can maintain transcranial intensities while minimising peripheral somatosensory confounds.

#### 3.4.3. Near-field peaks

The amplitude of near-field peaks in the scalp – sometimes caused by axial steering with commonly used annular arrays – significantly impacts peripheral somatosensory co-stimulation (*F* (1,24) = 37.9, *p* < 0.0001, ${\text{η}}_{\text{p}}^{2}=0.612$). Greater near-field peak amplitudes present at higher focal depths for this transducer (250-2 C H) resulted in more somatosensation (Fig. 5C). These sensations could be eliminated by minimising near-field peaks in the scalp through improved transducer manufacturing and/or using an appropriate combination of axial steering and coupling medium offset.

## 4. Discussion

In this pre-registered [54] study, we present evidence of peripheral somatosensory confounds during TUS in humans. A comprehensive understanding of the nature of these confounds and the conditions under which they arise is necessary to conduct well-controlled, robust, and minimally burdensome TUS research. Therefore, we systematically mapped the confound parameter space and demonstrated that somatosensory co-stimulation can be minimised by avoiding near-field peaks in the scalp, spreading energy across a greater area of the scalp, using ramped pulses, lowering ‘dose-sharpness’, and administering higher pulse repetition frequencies, higher fundamental frequencies, and lower doses. We also identify particle displacement as a putative biophysical driving force behind peripheral somatosensory confounds. With appropriate mitigation strategies, somatosensory co-stimulation can be minimised while maintaining meaningful TUS doses. Our findings lay the foundation for TUS parameter optimisation to enhance specificity and reliability in research and clinical settings.

### 4.1. Dose-response of the somatosensory confound

All participants experienced tactile, thermal, and painful sensations, with common descriptors including ‘buzzing’, ‘prickling’, ‘sharpness’, and ‘electric current’ (Fig. 2). Note that any noxious sensations are not caused by biological damage, and these sensations were not present for all protocols. The primary determinant of somatosensory confounds is dose, defined as the integral of intensity over the pulse train [70]. This definition also aligns with the term ‘exposure’, whereas a more precise account of dose (e.g., absorbed, equivalent, or effective) would consider interactions with biological tissues (see Nandi et al., 2025) [65]. Nonetheless, the broader term ‘dose’ is used here for simplicity. Higher doses amplify the somatosensory confound, both when the achieved by increasing intensity or pulse duration, suggesting that these modalities are, to some extent, interchangeable components of dose.

Without adequate controls, observed effects may be erroneously attributed to transcranial neuromodulation, while their causative origin lies in peripheral confounds. Indeed, such misinterpretations have already arisen in the context of the TUS auditory confound [14]. The challenge, then, lies in determining how best to minimise this confound without simply reducing dose, which risks compromising the intended neuromodulatory effects in the brain. Multiple strategies for addressing this challenge are discussed below.

### 4.2. Pulse shaping & temporal characteristics

Reducing ‘dose sharpness’ by delivering an equivalent dose with longer, lower-intensity pulses instead of shorter, higher-intensity pulses effectively minimises tactile co-stimulation, which is the most prominent somatosensory confound (Fig. 3). Note that there likely remains an absolute minimum intensity required for neuromodulation, and excessively high duty cycles may negate certain pulse repetition frequency (PRF) related effects [71,72]. Nonetheless, several studies demonstrate that keeping ‘dose sharpness’ low by increasing dose through longer pulse durations rather than through higher intensities also results in stronger TUS effects on the CNS [27,71–73]. Therefore, administering the same dose with a lower ‘dose sharpness’ can reduce tactile somatosensory effects while still evoking meaningful CNS neuromodulatory effects, and this approach thus constitutes one avenue for confound mitigation.

In addition, ramping the pulse envelope can effectively reduce somatosensory confounds by more than 50 % (Fig. 4A). Specifically, we show that 10 and 50 ms tapered cosine ramps significantly increase sensory thresholds. However, it is important to note that ramping inherently reduces dose by lowering the intensity integral in proportion to the ramp length. There may be a trade-off wherein the benefits of confound minimisation become outweighed by the reduction in dose beyond a given tipping point. In the present study, a 10 ms ramp duration offered the optimal balance: confounds were reduced by ~45%, while dose was only marginally reduced by 12.5%, compared to a square-wave envelope. Previous studies have demonstrated that effective CNS neuromodulation remains feasible with ramped pulses [8,27,74–76], thus supporting the viability of ramping for mitigation of somatosensory confounds, in addition to its well-established efficacy for auditory confounds [24,26,27,77].

Pulse repetition frequencies (PRFs) of 200 Hz and higher elicited ~30% less sensations than lower frequencies, suggesting that PRF can be tuned to minimise the somatosensory confound (Fig. 4B). Importantly, the dependence of co-stimulation magnitude on PRF suggests a relationship to endogenous neurophysiological firing rates [74,78]. For example, perhaps Type 1 rapidly adapting mechanoreceptors were preferentially activated in this study, given their sensitivity to the lower range of the applied PRFs [79,80]. Different PRFs may elicit distinct effects in relation to mechanoreceptive frequency sensitivity and firing rates [36]. Although higher PRFs can reduce co-stimulation, they are associated with stronger auditory confounds and offer limited opportunities for ramping, which our results suggest could be a more effective mitigation approach. Nonetheless, PRF can be considered as one of several parameters that can be optimised, with higher PRFs remaining capable of eliciting convincing neuromodulatory effects [11,81].

Over longer timescales, somatosensory co-stimulation may develop progressively. Indeed, we show that longer pulse train durations, commonly used in offline TUS protocols, can elicit a gradual buildup of co-stimulation. Dividing these protocols into segments could mitigate this effect. For example, intermittent TUS protocols, such as the ‘accelerated theta-burst’ protocol, successfully incorporate 30-min intervals between pulse trains [82].

In contrast, there was no inter-trial cumulation of co-stimulation throughout this online experiment. Bayesian analyses strongly indicated equivalence in VAS ratings for identical protocols delivered successively or interspersed throughout trials, and between consecutive sets of protocols. The absence of inter-trial cumulation validates the feasibility of trial-based study designs where conditions are repeated over time.

### 4.3. Transducer-specific parameters

Intensity peaks in the transducer near-field can significantly contribute to peripheral co-stimulation and should be circumvented. These near-field peaks are common for the transducers widely employed in human TUS research, such as the annular arrays used in this study. Our findings show that, as the focal depth for our annular array transducer increased, so did both near-field peaks and somatosensory confounds (Fig. 5C, lower panel). For currently available transducer designs, it is crucial to consider the transducer-specific focal depths at which these peaks occur and ensure they do not overlap with peripheral nerve structures. This can be achieved by selecting an appropriate combination of focal depth and coupling medium thickness.

The spread of intensity across the scalp can also be exploited to minimise confounds. Specifically, we show that a 33.18 cm^2^ aperture area transducer evoked substantially less somatosensation than a 15.9 cm^2^ aperture area transducer delivering the same integrated total intensity in the scalp. Multi-transducer constellations and hemispheric arrays [7–9,83,84] can therefore also be expected to circumvent peripheral somatosensory confounds, ostensibly up to very high transcranial intensities, without a substantial impact on CNS neuromodulation.

Finally, higher fundamental frequencies (500 kHz) produced fewer somatosensory confounds than lower frequencies (250 kHz), even when integrated maximum and total intensities in the scalp were equalised. Importantly, the intensity in the brain was higher for 500 kHz stimulation, thus demonstrating that higher frequencies maintain their advantage in reducing somatosensory confounds even if considering differences in acoustic transmission. Reduced co-stimulation compared to 250 kHz could be influenced by factors including smaller near-field volumes (5x) and potential destructive interference in the scalp caused by reflections off the skull for 500 kHz (λ = 3 mm) but not for 250 kHz (λ = 6 mm) where wavelength more closely matches scalp thickness. However, where these factors can be controlled, for example during ultrasound of the fingertip, lower frequencies are also more effective in eliciting sensations [34–37,42,51,51,52]. While we cannot assert whether the primary or secondary characteristics of fundamental frequency drive our results, there undoubtedly remains a practical advantage of higher frequencies for confound mitigation. Importantly, we also observed a dose-response effect at 500 kHz, highlighting that increasing frequency is not a one-stop solution for somatosensory confounds.

### 4.4. Particle displacement as a primary biophysical driving force underlying peripheral somatosensation

The systematic parameter optimisation approach taken here presents a valuable opportunity to infer the primary biophysical effects that drive neuromodulatory efficacy by leveraging known parameter-biophysics relationships. This approach is one of the few viable methods for making such inferences in healthy human populations. However, limited conclusions can be drawn based on this study alone, and it remains an open question whether peripheral biophysical parameter-effect relationships will translate to the central nervous system.

Putative biophysical mechanisms include acoustic cavitation, particle displacement, acoustic radiation force (ARF), and their respective strain. Cavitation is an unlikely mechanism, as somatosensory co-stimulation occurred well below the cavitation threshold, and empirically observed cavitation is not related to evoked sensations during ultrasound directly focused at the PNS [34]. ARF is dependent on absorption and scales with *f_0_* and intensity [70]. However, we observed effects that were inversely related to *f_0_* and scaled linearly with pressure. The observation of stronger effects at lower fundamental frequencies that scale with pressure implicate particle displacement over ARF as the primary driving force behind peripheral somatosensory co-stimulation, in line with findings from peripherally targeted ultrasound [34–36].

This preliminary evidence for particle displacement as a primary biophysical mechanism does not preclude a complementary role of ARF (strain). In fact, ARF may particularly contribute to tactile sensations, which were most pronounced at a higher ‘dose sharpness’ and in absence of ramping. Here, the sharper (temporal gradient of) ARF displacement could resemble a light ‘tap’ that peripheral mechanoreceptors are highly sensitive to. Nonetheless, the increase in sensations with longer pulse durations across all modalities indicates that a temporally stable component – either sustained ARF displacement or, more likely, the sign-alternating ultrasonic stimulus itself – also contributes to these effects.

It is likely that multiple biophysical effects of ultrasound work in tandem to drive (peripheral) neuromodulation. Future parametric studies can help us converge on a unified theory of key biophysical mechanisms. This pursuit will be critical to identify the principal biophysical effects in PNS and CNS neuromodulation, thus allowing for optimisation of TUS efficacy in the CNS while minimising effects on the PNS. For instance, if ARF were ultimately identified as a central CNS mechanism, as has been suggested [34,45,85,86], then adaptation towards higher sub-MHz frequencies and ARF interference setups [87] would become strong avenues to maximise effective dose.

### 4.5. Limitations

This study deliberately applied TUS in a manner expected to cause stronger somatosensory co-stimulation to avoid floor effects and have sufficient sensitivity to detect the effects of changes in stimulation parameters. Specifically, we used lower frequencies (250 kHz), included near-field intensity peaks, and stimulated through the temporal window where somatosensory co-stimulation is more pronounced. We note that stimulation over alternative scalp sites with a lower density of peripheral nerves, such as sites used for targeting the primary motor cortex or cingulate cortex, will result in substantially fewer somatosensory effects. However, the reported confound–parameter relationships likely generalise across sites, and we expect that the proposed mitigation approaches will therefore fully eliminate peripheral somatosensory co-stimulation over these less sensitive sites. Additionally, participants focused on co-stimulation, rather than on a cognitive task that might have reduced confound salience, though this does not negate risks of cueing or ineffective blinding. By operating under conditions that amplify confounds, we reliably mapped parameter-confound relationships, thereby providing actionable strategies to minimise co-stimulation that will also hold at lower confound levels.

In the present study we characterise peripheral somatosensory confounds and their relationship with stimulation parameters, thus laying the foundation for future studies to directly assess the efficacy of (in) active control conditions or alternative interventions such as topical anaesthetics in blinding participants to stimulation. Topical anaesthetics are unlikely to fully ameliorate (painful) somatosensory co-stimulation considering its primary effects on C-fibres [37] and its limited efficacy to this end for transcranial electric stimulation [88]. Future studies should directly quantify the efficacy of various controls such as defocusing or (in)active control in blinding the somatosensory confound when it cannot be eliminated.

### 4.6. Somatosensory confound mitigation strategies

**Fig. 6.**
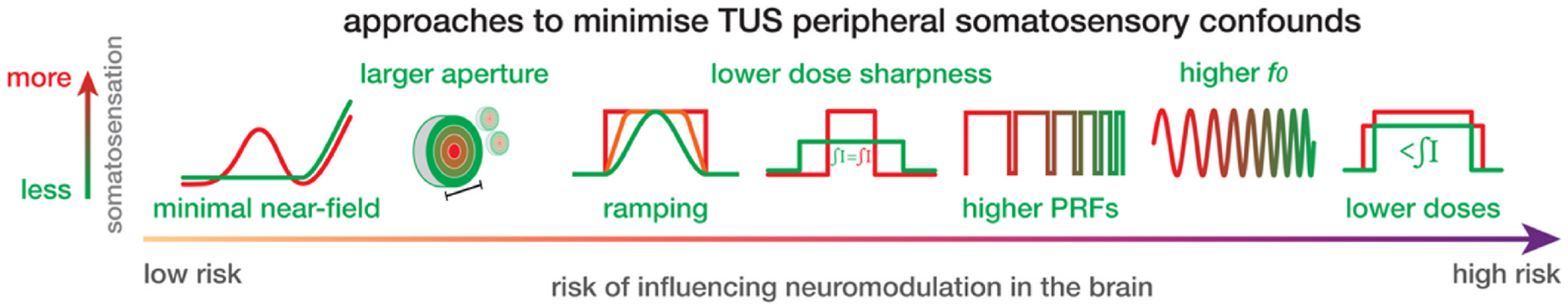
Approaches to minimise TUS peripheral confounds in order of risk of influencing neuromodulation in the brain. Conditions under which somatosensory co-stimulation is less pronounced are illustrated in green.

We propose the following workflow to minimise and control for somatosensory confounds in human TUS research. First, the likelihood of peripheral confounds should be assessed during study piloting. If somatosensory co-stimulation is likely, researchers can determine whether transducer-specific characteristics like near-field intensity peaks and energy dispersion in the scalp can be adapted to circumvent confounds. These interventions will have little-to-no impact on CNS neuromodulatory efficacy. Next, pulsing parameters can be optimised by introducing ramping and decreasing ‘dose sharpness’. If somatosensory confounds persist, researchers can consider adjusting fundamental frequency, PRF, or dose itself (Fig. 6). However, undesired impact on CNS neuromodulation should be carefully considered for these manipulations. For example, fundamental frequency selection should holistically balance the required target spatial resolution, as well as the relevant safety metric boundaries, desired primary biophysical effects, and practical constraints [59,70,71]. Future studies could map peripheral confounds and CNS neuromodulatory efficacy in parallel by leveraging emerging testbeds for online TUS effects, such as neural engagement metrics or biased visual choice behaviour during stimulation of the lateral geniculate nucleus [8] or frontal eye fields [89]. This would enable direct empirical quantification of the optimal balance between confound minimisation and effective brain stimulation.

Building on the characterisation of somatosensory co-stimulation presented in this study, future research should further assess the efficacy of control conditions that sufficiently mimic somatosensory confounds when they cannot be fully alleviated. Common sound-only sham conditions will not sufficiently reproduce somatosensory co-stimulation. Therefore, active or inactive control stimulation sites are preferred, where this limitation is addressed by precisely replicating auditory and somatosensory confounds without delivering effective dose. Defocusing the transducer may also be an effective control technique, though this is not possible for all transducers. Furthermore, care should be taken that there is a similar intensity profile in the scalp during verum and control conditions. Using these controls, we can make substantiated inferences on direct neuromodulatory effects, even when peripheral confounds are present.

## 5. Conclusion

Managing somatosensory confounds is critical to minimise participant burden and ensure valid and replicable findings as TUS research progresses toward higher doses, more frequent transducer placement at the sensitive temples of the head, and smaller transducers. This study characterises the range of somatosensory co-stimulation experienced during TUS and identifies effective mitigation strategies. These include reducing near-field intensity peaks in the scalp, dispersing energy across the scalp, ramping the pulse envelope, and lowering ‘dose sharpness’. Higher pulse repetition frequencies, higher fundamental frequencies, and lower doses further minimise these effects. Where confounds cannot be fully resolved, robust control conditions, such as (in)active controls that replicate auditory and somatosensory confounds, are essential to isolate direct neuromodulatory effects. By adopting these strategies, researchers can enhance the reliability of TUS research and accelerate effective ultrasonic neuromodulation in scientific, commercial, and clinical domains.

## Supplementary Material

1

## Data Availability

Data and code to reproduce the results reported in this study are available here: https://doi.org/10.5281/zenodo.14052159.

## Appendix A. Supplementary data

Supplementary data to this article can be found online at https://doi.org/10.1016/j.brs.2025.06.009.
